# Evaluation of the Potential Cytoprotective Effect of Melatonin in Comparison with Vitamin E and Trolox against Cd^2+^-Induced Toxicity in SH-SY5Y, HCT 116, and HepG2 Cell Lines

**DOI:** 10.3390/ijms25158055

**Published:** 2024-07-24

**Authors:** Rosanna Mallamaci, Alexia Barbarossa, Antonio Carrieri, Daniela Meleleo, Alessia Carocci

**Affiliations:** 1 Department of Biosciences, Biotechnologies and Environment, University of Bari “Aldo Moro”, 70125 Bari, Italy; rosanna.mallamaci@uniba.it; 2 Department of Pharmacy–Pharmaceutical Sciences, University of Bari “Aldo Moro”, 70125 Bari, Italy; alexia.barbarossa@uniba.it (A.B.); alessia.carocci@uniba.it (A.C.); 3 Department of Science of Agriculture, Food, Natural Resources and Engineering, University of Foggia, 71122 Foggia, Italy

**Keywords:** antioxidants, cytotoxicity, heavy metal, oxidative stress, human cell lines

## Abstract

Cadmium (Cd) toxicity poses a significant threat to cellular health, leading to oxidative stress and cell damage. Antioxidant agents, particularly those of natural origin, have been studied as a potential alternative for mitigating heavy metal toxicity. This study aimed to evaluate the cytoprotective effects of the antioxidant melatonin (MLT) in comparison with Vitamin E (VitE) and Trolox against Cd^2+^-induced cellular toxicity. The MTT assay was employed to assess cell viability in neuronal SH-SY5Y, colorectal HCT 116, and hepatic HepG2 cell lines. The results showed that all three antioxidants offered some level of protection against Cd toxicity, with Vitamin E proving to be the most effective. MLT also demonstrated a substantial cytoprotective effect, especially at the highest Cd concentration of 30 µM. These findings suggest that MLT, alongside Vit E and Trolox, could be valuable in mitigating the detrimental effects of Cd exposure by reducing the oxidative stress in these cellular models.

## 1. Introduction

**Figure 1:**
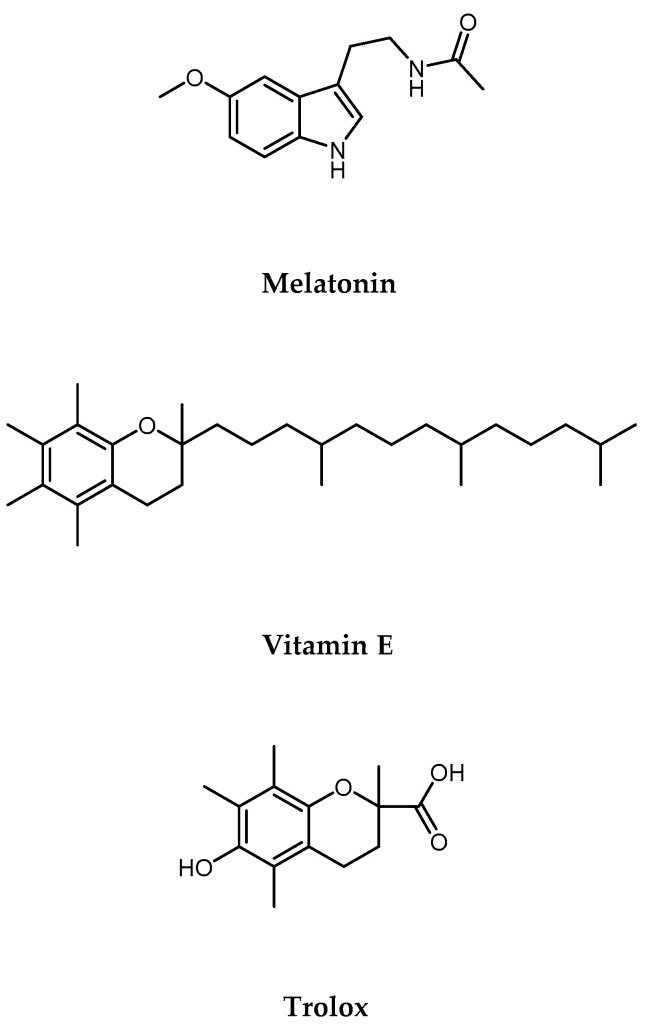
Melatonin, Vitamin E, and Trolox structures.

Cadmium (Cd) is a heavy metal pollutant that is commonly found in the environment because of industrial activities, mining, and agricultural practices [1,2]. Human activities like burning fossil fuels, processing metal ores, and burning waste add Cd to the environment. When sewage sludge is added to farm soil, it transfers cadmium to plants, affecting the food chain [3] and leading to its buildup in human organs. Cigarette smoke is a significant source of Cd exposure [4]. Among different Cd compounds, cadmium oxide (CdO) is primarily absorbed through the respiratory tract, while only a small portion (1–10%) of the oral dosage of cadmium chloride (CdCl_2_) is absorbed through the gut [5]. Once in the body, Cd is transported through erythrocytes and albumin into the bloodstream, where it accumulates primarily in the kidneys, liver, and intestines [6]. The body eliminates Cd slowly, primarily through the kidneys, urine, saliva, and breast milk during lactation [7]. Extensive scientific literature highlights its harmful effects on human health, with potential illnesses including abnormal spermatogenesis and infertility [8], altered lung function [9], hormonal imbalance [10], renal toxicity [11], cardiovascular diseases [12], inflammatory conditions [13], and even cancer [14,15]. Long-term exposure to Cd increases the risk of lung, prostate, and kidney cancers. Cd acts as a carcinogen by promoting DNA damage, inhibiting DNA repair, and facilitating tumor growth [16]. Studies have shown that Cd can cause cellular transformation and carcinogenesis in human colon cell lines [17]. Additionally, Cd toxicity has been linked to neurodegenerative diseases such as Alzheimer’s and Parkinson’s, although the mechanisms involved are complex [18]. One important mechanism involves the induction of oxidative stress in the central nervous system. Cd has been found to produce reactive oxygen species (ROS) through various pathways, which include the inhibition of antioxidant enzymes like superoxide dismutase and catalase from functioning properly and disrupting mitochondrial function [19]. Additionally, Cd can activate neuroinflammation, which involves the activation of microglia and the release of pro-inflammatory cytokines such as interleukin-1β and tumor necrosis factor-alpha. Prolonged neuroinflammation leads to neuronal injury and death, worsening the progression of neurodegenerative diseases [20]. The hepatotoxic effects of Cd are primarily caused by the generation of oxidative stress within hepatocytes. Cd exposure leads to the production of reactive oxygen species (ROS), which overwhelms the antioxidant defense mechanisms of the liver [21]. The intricate relationship between Cd toxicity and oxidative stress-induced damage across various organs highlights the importance of researching natural substances with antioxidant properties [22,23]. Melatonin (MLT), a hormone primarily known for its role in regulating circadian rhythms, has emerged as a potent antioxidant with promising cytoprotective properties [24,25]. Studies in cellular models have demonstrated MLT’s ability to scavenge free radicals, mitigate oxidative stress, and preserve cellular integrity in the face of toxic insults [26,27]. The aromatic indole ring of MLT plays a pivotal role in buffering and scavenging ROS and nitrogen species (RNS) [28]. This antioxidant function is augmented by the subsequent antioxidant activity of its reaction products and metabolites in a cascade-like manner. Moreover, MLT exerts its protective effects by upregulating endogenous antioxidant enzymes, including superoxide dismutase, catalase, glutathione peroxidase, and glutathione reductase. Additionally, MLT can decrease the activation of the transcription factor nuclear factor kappa B (NF-κB), leading to a reduction in inflammatory mediators such as cytokines, enzymes, and adhesion molecules [29]. In addition, some studies in the literature report the ability to counteract Cd toxicity in different tissue districts [30,31]. Vitamin E (Vit E) and its water-soluble analog, Trolox, have been extensively studied for their ability to protect against oxidative stress-induced damage. These compounds exert their protective effects by scavenging free radicals and stabilizing cell membranes, thereby mitigating oxidative damage. Additionally, Vitamin E and Trolox can enhance the activity of endogenous antioxidant enzymes, such as superoxide dismutase and catalase, further bolstering cellular defenses against oxidative stress [32,33,34,35]. Their antioxidant action has been demonstrated in various cellular models [36,37,38] as well as in vivo [39,40]. Given the powerful antioxidant activity of Vitamin E, studies have investigated its action in counteracting Cd toxicity [41,42]. Considering the critical role of oxidative stress in Cd-induced toxicity, which, in turn, contributes to cellular damage and dysfunction and, consequently, leads to the pathogenesis of various diseases, antioxidants have emerged as a promising therapeutic approach to mitigate the detrimental effects of Cd exposure. In this study, our objective was to assess the protective effects of MLT against Cd-induced cellular toxicity in comparison to VitE and Trolox (Figure 1), whose antioxidant properties are well documented. To address this issue, the human hepatocellular carcinoma (HepG2) cell line was used as a corroborated cellular model for Cd toxicity studies [43]. Furthermore, the neuroblastoma cell line SH-SY5Y and the human colorectal carcinoma cell line HCT 116 were selected as cellular models for neurotoxicity and cellular responses to toxic agents, respectively [44,45,46,47]. Hence, herein, we report the results of comparative studies of the cytoprotective effect of these three antioxidants (MLT, VitE, and Trolox) on Cd toxicity.

## 2. Results

### 2.1. Effect Cd^2+^, MLT, Vit E, and Trolox on Cell Viability

**Figure 2:**
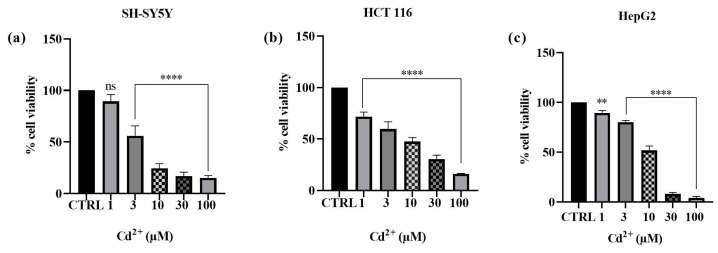
Effects of Cd^2+^ (1–100 µM) on SH-SY5Y (**a**), HCT 116 (**b**), and HepG2 (**c**) cell viability after 24 h of exposure. Results are shown as mean ± standard deviation (SD) *(n* = 3). Significant differences versus the control (CTRL): non-significant differences (ns, *p* > 0.05), ** *p* < 0.01, and **** *p* < 0.0001.

Cytotoxicity assays were conducted to assess how Cd^2+^ impacts the viability of human SH-SY5Y neuronal cells, HCT 116 colon cancer cells, and HepG2 hepatoma cells. The results obtained are depicted in Figure 2 (see Supplementary Materials, Table S1). The effects of the metal under investigation on cell viability were assessed by means of the MTT assay. After 24 h of exposure to different concentrations of Cd^2+^, a dose-dependent cytotoxic effect was observed in all the cell lines studied. The cell line that proved to be most sensitive to Cd^2+^ toxicity was found to be the hepatocarcinoma cell line. Indeed, the cell survival rate dramatically declined during 24 h at doses of 1, 3, 10, 30, and 100 µM, decreasing from 89.4 to 80.2, 51.7, 8.2, and 4.2%, respectively. Conversely, for the neuroblastoma cell line, cell viability decreased from 89.2%, at the lowest concentration of 1 µM, to 15.2%, at the maximum concentration of 100 µM. A similar trend in the results was observed in Cd^2+^-induced toxicity towards the colon cancer cell line, where cell viability decreased from 71.8% (at a concentration of 1 µM) to 16.2% (at a concentration of 100 µM). The findings obtained with this first set of experiments enabled us to select the Cd^2+^ concentrations that notably reduced viability for assessing the potential protective effect of the tested substances, i.e., MLT, VitE, and Trolox. Consequently, the concentrations of 10 µM and 30 µM were selected for subsequent experiments because, among all three cellular models, they were identified as the concentrations capable of impacting cell viability without entirely compromising the potential cytoprotective activity of the compounds under investigation, as these concentrations of Cd^2+^ did not induce complete cell death [24].

**Figure 3:**
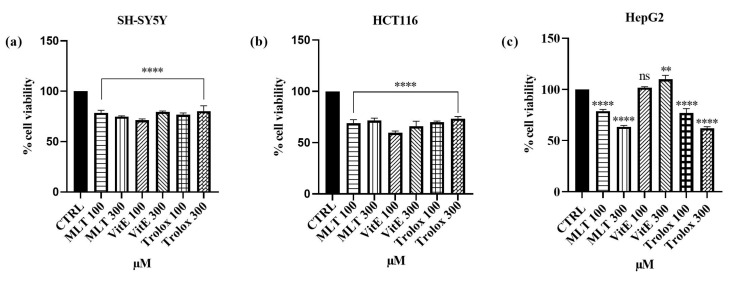
Effects of MLT, VitE, and Trolox (100–300 µM) on SH-SY5Y (**a**), HCT 116 (**b**), and HepG2 (**c**) cell viability after 24 h of exposure. Results are shown as mean ± standard deviation (SD) *(n* = 3). Significant differences versus the control (CTRL): non-significant differences (ns, *p* > 0.05), ** *p* < 0.01, and **** *p* < 0.0001.

Furthermore, to explore the effect of MLT, VitE, and Trolox on the SH-SY5Y, HCT 116, and HepG2 cell lines, they were exposed to concentrations of 100 and 300 micromolar of the compounds for a 24-h incubation period. The results are depicted in Figure 3 (see Supplementary Materials, Table S2). Regarding SH-SY5Y cells, a 24-h incubation period with MLT at concentrations of 100 and 300 µM induced viabilities of 78.5% and 74.9%, respectively. At the same concentrations, slightly lower cell viabilities were observed for HCT 116 cells, with percentages of 69.9% and 71.6%. Regarding the hepatocarcinoma cells, incubation with 100 µM of MLT resulted in a viability of 78.8%, while incubation with 300 µM slightly reduced viability, resulting in 63.6%. VitE, on the other hand, exhibited distinct impacts on the three cell lines. Indeed, 24 h of incubation with Vitamin E resulted in a cell viability percentage of 71.2% (100 µM) and 79.6% (300 µM) in SH-SY5Y cells. Conversely, in HCT 116 cells, after exposure to concentrations of 100 µM and 300 µM of Vitamin E, a decrease in cell viability compared with the control was observed, with viability percentages of 59.7% and 66%, respectively. Instead, a pro-proliferative effect was observed after incubating HepG2 cells with VitE. In fact, the cell viability values were 101.7% (VitE concentration of 100 µM) and 109.8% (VitE concentration of 300 µM). The effects of Trolox on cell viability were found to be more favorable for neuroblastoma cells, as a viability value of 76.7% was recorded at 100 µM, which then increased to 80.3% at a concentration of 300 µM. On the other hand, for HCT 116 cells, cell viability after exposure to Trolox increased from 69.9% (100 µM concentration) to 73.5% (300 µM concentration), while for HepG2 cells, it decreased from 77% to 62.2% for concentrations of 100 and 300 µM, respectively.

### 2.2. Effect of MLT, VitE, and Trolox on Cd^2+^-Induced Cytotoxicity

**Figure 4:**
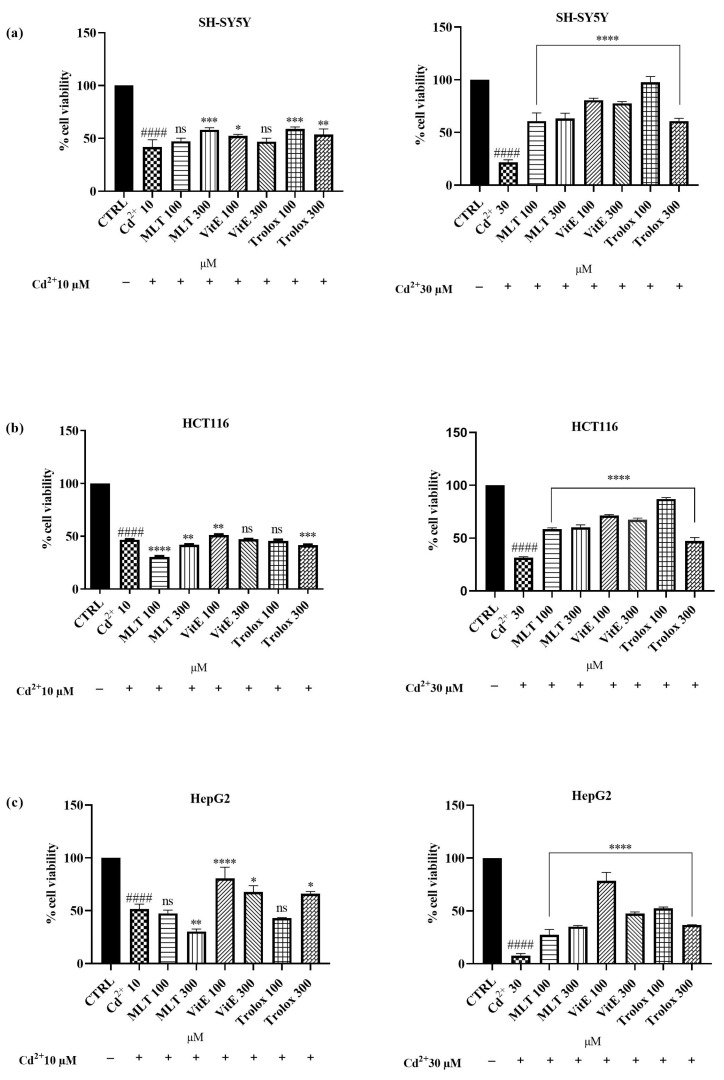
Effect of simultaneous treatment with Cd^2+^ (10 or 30 µM) and MLT, VitE, and Trolox (100 or 300 µM) on SH-SY5Y (**a**), HCT 116 (**b**), and HepG2 (**c**) cell viability after 24 h of exposure (MTT assay). Data are expressed as mean ± standard deviation (SD) (*n* = 3). Significant differences versus the control (CTRL): #### *p* < 0.0001; non-significant differences (ns, *p* > 0.05), * *p* < 0.05, ** *p* < 0.01, *** *p* < 0.001, and **** *p* < 0.0001 as compared with Cd^2+^ 10 or 30 µM alone.

After confirming that the tested concentrations of MLT, VitE, and Trolox did not exhibit significant toxicity towards the SH-SY5Y, HCT 116, and HepG2 cell lines, we investigated the potential cytoprotective effects against cell damage induced by exposure to Cd^2+^ at concentrations of 10 and 30 µM. To evaluate this protective effect, cells were co-treated with MLT, VitE, and Trolox (100, 300 µM) and Cd^2+^ (10, 30 µM) for an incubation period of 24 h. The obtained results are reported in Figure 4 (see Supplementary Materials, Tables S3 and S4). Interestingly, among the three cellular lines used, MLT was able to significantly counteract Cd^2+^ toxicity (10 µM) towards the SH-SY5Y cell line. Cellular viability increased from a percentage of 41.8% (Cd^2+^-only treated cells) to 47.3% (cells treated with 100 µM of MLT) up to 58.2% (cells treated with 300 µM of MLT). The cytoprotective effect of MLT against the same cell line was found to be even more pronounced against the toxicity induced by the higher concentration of Cd^2+^, equal to 30 µM. Indeed, cell viability increased nearly threefold, rising from 21.7% (cells exposed to Cd^2+^ alone) to as high as 60.9% and 63.3% when the cells were simultaneously treated with Cd^2+^ (30 µM) and MLT at concentrations of 100 µM and 300 µM, respectively. In the same cellular model, however, a milder cytoprotective effect was obtained with VitE combined with Cd^2+^ at a concentration of 10 µM. In fact, cellular viability following co-treatment with VitE at 100 µM and 300 µM was found to be 52.4% and 47%, respectively. Surprisingly, co-treatment of neuroblastoma cells with Cd^2+^ at a concentration of 30 µM and VitE (100 µM and 300 µM) significantly counteracted the cytotoxic effects of Cd^2+^, exhibiting a viability of 80.7% and 77.7%, respectively. Similarly, Trolox showed moderate cytoprotective efficacy towards SH-SY5Y treated with the lower concentration of Cd^2+^. However, a remarkable effect was observed when combining Cd^2+^ at a concentration of 30 µM with Trolox at a concentration of 100 µM. In fact, a significant increase was observed, rising from 21.7% (cells treated only with Cd^2+^) to 97.7% (simultaneous treatment with Trolox 100 µM and Cd^2+^). Regarding the HCT 116 cells, no cytoprotective effect was observed with MLT against Cd-induced toxicity at a concentration of 10 µM. Conversely, when co-treating HCT 116 cells with Cd at a concentration of 30 µM and MLT at concentrations of 100 µM and 300 µM, there was an approximately twofold increase in cellular viability. The percentage of viability increased from 31.6% (cells treated only with Cd^2+^) to approximately 60% (co-treatment with Cd^2+^ and MLT 100 µM and 300 µM). In the same cellular model, VitE was successful in counteracting the cytotoxicity induced by Cd^2+^ at a concentration of 30 µM. Cellular viability increased from 31.6% to approximately 70% when cells were treated with Cd^2+^ and both the concentrations of VitE used in the test. For Trolox, the most encouraging effect was achieved by combining its concentration of 100 µM with Cd^2+^ 30 µM, resulting in cellular viability reaching a percentage value of 87% following co-treatment. Even towards the hepatocellular carcinoma cell line, MLT managed to improve the cytotoxic effect only following co-treatment with Cd^2+^ at a concentration of 30 µM. Cellular viability, in this case, increased from 7.6% (Cd^2+^-only treated cell) to 27.3% (co-treatment with MLT 100 µM) and up to 35.2% (co-treatment with MLT 300 µM). However, even in this case, the most significant cytoprotective effect was obtained by VitE, especially when used at a concentration of 100 µM. Indeed, the exposure of HepG2 cells to only Cd^2+^ (10 µM) resulted in a vitality of 51.7%, while simultaneous treatment with VitE 100 µM led to a cellular growth of 80.7%. Even more significant was the effect against the toxicity induced by the higher concentration of Cd^2+^, which caused significant cell death in HepG2, with cellular vitality at 7%, while after co-treatment with VitE 100 µM, the number of viable cells was 78.6%. No protective effect was found for Trolox at a concentration of 100 µM against Cd^2+^ 10 µM. However, a slight increase in cell viability (about 15% compared with cells treated only with Cd^2+^) was observed when using Trolox at a concentration of 300 µM. The results obtained instead showed a better activity of Trolox against the toxicity induced by the higher concentration of Cd^2+^, 30 µM, on HepG2 cells. However, even in this case, the concentration of Trolox that demonstrated a more pronounced cytoprotective effect was 100 µM. In fact, the percentage of viable cells increased by 44.8% for those exposed to the simultaneous treatment of Cd^2+^ with Trolox 100 µM and by 29.2% for those co-treated with Cd^2+^ and Trolox 300 µM, compared with cells exposed solely to Cd^2+^. Taken together, these results suggest that although all three antioxidant molecules were able to improve Cd^2+^ toxicity, VitE is the most effective substance in counteracting these effects, followed by Trolox and, finally, MLT.

### 2.3. ROS Scavenging Effects of MLT, VitE, and Trolox against Cd^2+^- and H_2_O_2_-Induced Oxidative Stress

**Figure 5:**
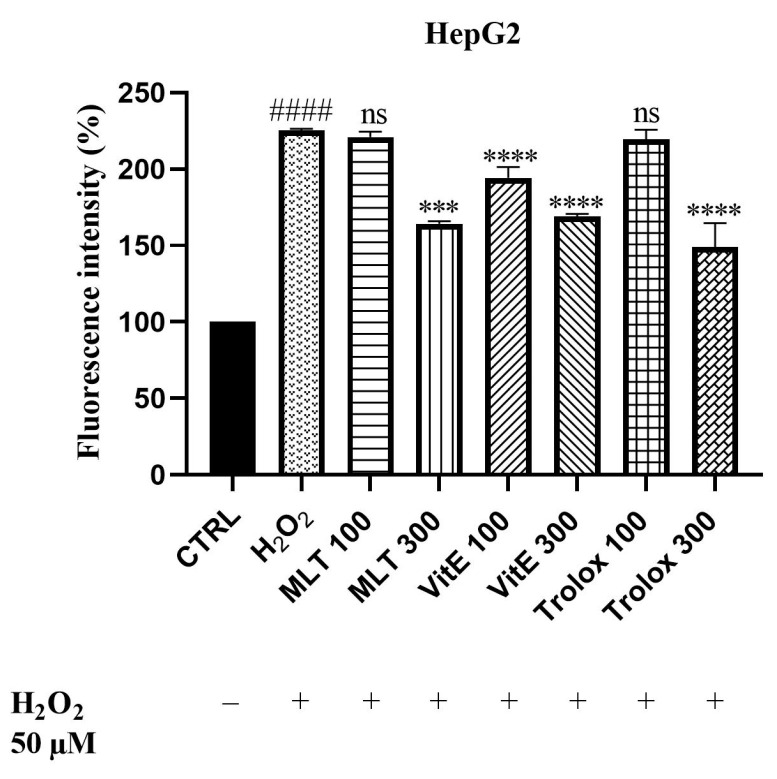
DCFH oxidation in HepG2 cells after 6 h of incubation with MLT, VitE, and Trolox (100 or 300 µM) in the presence of hydrogen peroxide (H_2_O_2_). Data are expressed as mean ± standard deviation (SD) (*n* = 3). Significant differences versus the control (CTRL): #### *p* < 0.0001; non-significant differences (ns, *p* > 0.05), *** *p* < 0.001, and **** *p* < 0.0001 as compared with H_2_O_2_.

Since oxidative stress is one of the main mechanisms of Cd^2+^-induced toxicity, we aimed to explore the potential ability of MLT, VitE, and Trolox to reduce oxidative stress triggered by this heavy metal. The experiment was performed in HepG2 cells since they have an enhanced oxidative metabolism that causes cellular oxidative stress and/or generates reactive metabolites. It is therefore reasonable to assume that HepG2 cells are suitable for studying protection against oxidative and cytotoxic effects, if any. The potential antioxidant activity was evaluated in vitro using the 2′,7′-dichlorodihydrofluorescein diacetate (DCFH-DA) cell-based assay. The test was based on measuring the reducing effect of the compound against oxidation of 2′,7′-dichlorodihydrofluorescein (DCFH) to the fluorescent probe of 2′,7′-dichlorofluorescein (DCF). At first, we determined the ability of MLT, VitE, and Trolox to protect our cellular model from the ROS production induced by H_2_O_2_ (Figure 5; see Supplementary Materials, Table S5), which was used at a concentration of 50 µM. The results demonstrated that MLT, VitE, and Trolox were able to reduce the production of ROS generated by H_2_O_2_ in a dose-dependent manner. The results indicate that MLT at a concentration of 100 µM only slightly decreased ROS production. In contrast, a significant reduction of 61% was recorded following cell exposure to a concentration of 300 µM. VitE, on the other hand, effectively countered oxidative stress in the cellular model at both concentrations tested. The most pronounced effect was observed with Trolox, which, at a concentration of 300 µM, managed to reduce H_2_O_2_-induced ROS production by 76%.

**Figure 6:**
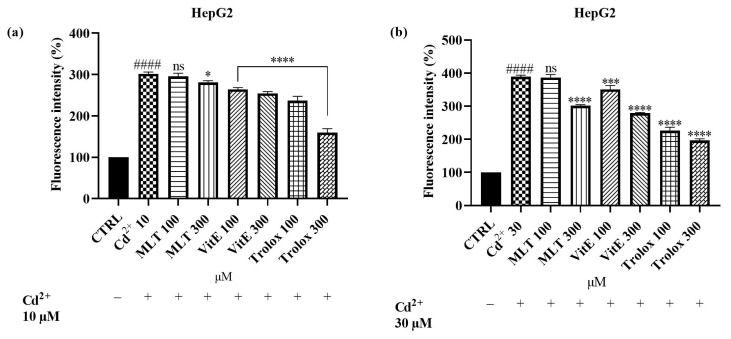
DCFH oxidation in HepG2 cells after 6 h incubation with MLT, VitE, and Trolox (100 or 300 µM) in the presence of Cd^2+^ 10 µM (**a**) and Cd^2+^ 30 µM (**b**). Data are expressed as mean ± standard deviation (SD) (*n* = 3). Significant differences versus the control (CTRL): #### *p* < 0.0001; non-significant differences (ns, *p* > 0.05), * *p* < 0.1, *** *p* < 0.001, and **** *p* < 0.0001 as compared with Cd^2+^ (10 or 30 µM).

Once we demonstrated the protective power of MLT, VitE, and Trolox against H_2_O_2_-induced oxidation, we evaluated the same effect against Cd^2+^-induced ROS production, used at concentrations of 10 and 30 µM (Figure 6; see Supplementary Materials, Tables S6 and S7). As in our previous experiments, the MLT, VitE, and Trolox compounds were more effective in counteracting the high concentration of Cd^2+^ (30 µM). Specifically, the simultaneous administration of MLT with Cd^2+^ (10 µM) reduced ROS production by only 20% at the highest MLT concentration used, whereas the same concentration co-administered with 30 µM Cd^2+^ reduced fluorescence by 87%. A significant protective effect against ROS generation induced by 30 µM of Cd^2+^ was observed with VitE. The simultaneous administration of VitE at 300 µM with Cd^2+^ (30 µM) resulted in a marked decrease in oxidative stress, amounting to 110%. The most encouraging effects were once again obtained with Trolox, which was effective in counteracting oxidative stress induced by both 10 µM and 30 µM of Cd^2+^. A pronounced dose-dependent effect was observed with Trolox; fluorescence intensity was reduced by 64% and 141% following treatment with 100 µM and 300 µM Trolox, respectively, and Cd^2+^ (10 µM). The same concentrations of Trolox reduced oxidative stress generated by the higher concentration of Cd^2+^ by 162% and 192%, respectively.

## 3. Discussion

The discussion surrounding heavy metal toxicity underscores its profound impact on human health, with Cd being one of the most concerning heavy metals because of its ubiquitous presence in the environment and its detrimental effects on various organs and physiological processes [48]. Oxidative stress induced by this xenobiotic is one of the main mechanisms responsible for liver, gut, and neurodegenerative diseases [49]. By binding to mitochondria, even at low concentrations, Cd can impede both cellular respiration and oxidative phosphorylation, further impacting cellular function and viability. Cd toxicity, connected with oxidative stress, manifests through several mechanisms including the following: depletion of reduced glutathione (GSH), binding sulfhydryl groups with proteins, and stimulating the production of reactive oxygen species (ROS) such as superoxide ions, hydrogen peroxide, and hydroxyl radicals [50]. Additionally, Cd inhibits the activity of key antioxidant enzymes, including catalase, manganese-superoxide dismutase, and copper/zinc-dismutase [51]. Metallothionein, a zinc-binding protein rich in cysteine, serves as a potent free-radical scavenger, effectively neutralizing hydroxyl and superoxide radicals. Cells containing metallothioneins demonstrate resistance to Cd toxicity, whereas those lacking the ability to synthesize metallothioneins are more susceptible to Cd-induced damage [52]. Furthermore, Cd modulates cellular calcium levels and influences the activities of caspases and mitogen-activated protein kinases (MAPKs), indirectly triggering apoptosis within affected cells. Recently, there has been significant interest among researchers in using antioxidants to prevent or delay the harmful effects caused by heavy metals. Studies have reported the ability of Vit E, a well-known naturally occurring antioxidant, and its hydro soluble analog, Trolox, to counteract cadmium-induced oxidative stress [53,54]. In addition, MLT, the main indolamine produced by the pineal gland, is small with high lipophilicity, crosses biological membranes easily, and reaches all sections of the cell [55]. Over the years, MLT has been recognized as a free radical scavenger with the ability to remove reactive oxygen species (ROS) including singlet oxygen, superoxide anion radical, hydroxyl radical, hydrogen peroxide, and lipid peroxides [56,57]. In addition, MLT administration ameliorates the pro-inflammatory state and oxidative stress in diabetic fatty rats [58]. Therefore, the aim of the following work was to compare the cytoprotective effects of the antioxidant substances MLT, Vit E, and Trolox against certain cellular models, including neuronal SH-SY5Y, colorectal HCT 116, and hepatic HepG2 cells.

### 3.1. Effect of Cd, MLT, VitE, and Trolox on Cell Viability

Cd was used to create an in vitro model of heavy metal-induced toxicity in SH-SY5Y, HCT 116, and HepG2 cells, aiming to replicate the effects of chronic heavy metal exposure in the corresponding tissues in vivo [43,59,60]. The MTT assay, a widely used method for determining cell viability, was conducted on these cell lines in the presence of varying concentrations of Cd^2+^. Our results demonstrated a dose-dependent cytotoxic effect of Cd^2+^ in all three cell lines tested, with the HepG2 cell line proving to be the most sensitive. The results obtained are in line with those previously reported. Indeed, previous studies have demonstrated the concentration-dependent cytotoxic effect of Cd on the HepG2 cell line, as well as its ability to activate genes involved in oxidative stress and to cause gradual mitochondrial membrane depolarization, thus increasing the release of pro-apoptotic mediators [61,62]. In HCT 116 cells, it has been demonstrated that Cd toxicity involves the activation of metalloproteases, serine proteases, and cysteine proteases [63]. The detrimental effect of Cd toxicity has also been demonstrated in SH-SY5Y cells alongside Cd’s capability to induce apoptosis through the PI3K-Akt-mTOR signaling pathway [64]. Therefore, the obtained findings allowed us to establish that the concentrations of 10 and 30 μM were the highest capable of affecting cell viability without entirely inducing cell death and therefore suitable to perform the other assays. Subsequently, the investigation of the effects of MLT, Vit E, and Trolox on cell viability was carried out on the three cell lines under study. The results showed a comparable effect on viability across the three lines for the three antioxidant compounds. Specifically, a slight decrease in viability was observed when the cells were treated for 24 h with concentrations of 100 and 300 µM of the three antioxidant compounds. Notably, an exception was found in HepG2 cells incubated with VitE, where cell viability increased.

### 3.2. Effect of MLT, VitE, and Trolox on Cd-Induced Cytotoxicity

In the second step of our study, we examined the effects of co-treatment with MLT, VitE, or Trolox on the three cell lines under investigation exposed to Cd. The antioxidants were used at concentrations of 100 and 300 μM, while Cd was administered at 10 and 30 μM. When the cell lines were exposed to the lower Cd concentration of 10 μM, the introduction of antioxidants led to slight or no significant improvements in cell viability. This suggests that the lower dose of Cd may not have been sufficiently cytotoxic to observe a substantial protective effect from the antioxidants. However, at the higher Cd concentration of 30 μM, we observed a notable increase in cell viability across all three cell lines when antioxidants were present. This indicates a more pronounced protective role of the antioxidants against higher levels of Cd-induced toxicity. Among the antioxidants tested, VitE consistently provided the most significant improvement in cell viability, outperforming both Trolox and MLT. The superior activity of VitE can be attributed to its well-documented role in protecting cell membranes from oxidative damage by scavenging free radicals [65]. The ability of VitE to integrate into lipid bilayers allows it to effectively prevent lipid peroxidation, a key mechanism of cellular protection [66]. Trolox, a water-soluble analog of VitE, also showed considerable protective effects but to a lesser extent than Vitamin E. MLT, while still effective, was the least potent of the three in counteracting Cd toxicity. The differential effectiveness of VitE and Trolox is consistent with previous studies showing that while both are potent antioxidants, the lipid-soluble nature of VitE allows it to provide more comprehensive protection in cell membrane-rich environments compared to its water-soluble counterpart, Trolox [67]. These findings highlight the differential efficacy of antioxidants in mitigating Cd-induced cytotoxicity, with VitE emerging as the most effective agent. The effectiveness of Trolox further supports the potent antioxidant capacity of Vitamin E derivatives. Although MLT was less effective compared with the other antioxidants, its contribution was still significant, underscoring its potential utility in reducing Cd toxicity. Interestingly, significant protective effects were observed specifically in the HepG2 cell line, which is derived from human liver carcinoma cells. This finding is noteworthy as it supports the potential use of antioxidant agents in counteracting Cd-induced hepatotoxicity. The HepG2 line’s response to antioxidant treatment underscores the liver’s vulnerability to Cd toxicity and the potential therapeutic value of antioxidants in protecting liver cells from such damage. Overall, this study emphasizes the importance of antioxidant choice in combating heavy metal toxicity and suggests that Vitamin E could be a particularly valuable agent in therapeutic strategies against Cd exposure. Further research is warranted to explore the mechanisms underlying these differential effects and to confirm the potential clinical applications of these findings.

### 3.3. ROS Scavenging Effects of MLT, VitE, and Trolox on Cd-Induced Oxidative Stress

Furthermore, we aimed to explore the protective effects of MLT, VitE, and Trolox, thus demonstrating their ability to reduce oxidative stress induced by Cd in HepG2 cells. The HepG2 cell line is a widely used cellular model for evaluating oxidative stress because these cells inherently have a high accumulation of ROS, making them particularly suitable for studying oxidative damage and the effectiveness of antioxidant compounds. Furthermore, the liver’s role in detoxification makes it a primary target for Cd accumulation and subsequent oxidative stress [43]. We first demonstrated that MLT, VitE, and Trolox were able to protect our model from ROS production induced by a stimulus with H_2_O_2_ in a dose-dependent manner. The same effect was demonstrated against Cd-induced ROS production. We observed that the compounds under study were more effective in reducing oxidative stress generated by the higher concentration of Cd. The best effect was obtained with Trolox, which significantly counteracted ROS production induced by both H_2_O_2_ and Cd. While some studies have explored the protective effects of individual antioxidants like VitE and MLT against Cd-induced oxidative stress [41,68], fewer studies have systematically compared the effectiveness of MLT, VitE, and Trolox in counteracting Cd-induced oxidative stress in a cellular model like the HepG2 cell line. This study contributes to the scientific literature by providing a comparative analysis of these antioxidants under identical experimental conditions, highlighting their relative efficacies and potential therapeutic applications. The findings emphasize the importance of antioxidant choice in combating heavy metal toxicity and suggest that VitE and its analogs could be particularly valuable in therapeutic strategies against Cd exposure.

## 4. Materials and Methods

### 4.1. Cytotoxicity and Cytoprotective Assays In Vitro

#### 4.1.1. Cell Culture

SH-SY5Y human neuroblastoma cells, HCT 116 human colon carcinoma cells, and HepG2 human hepatoma cells were used in this study and purchased from American-style culture collection (ATCC, Manassas, VA, USA). The cells were routinely grown in a monolayer culture in 25 cm^2^ flasks (Corning Inc., New York, NY, USA) and maintained in high glucose (4.5 gL^−1^) Dulbecco’s Modified Eagle Medium (DMEM) (PAN Biotech, Aidenbach, Germany) supplemented with 10% (*v*/*v*) fetal bovine serum (FBS) (PAN Biotech, Aidenbach, Germany) 1% penicillin–streptomycin solution (LONZA Bioscience, Walkersville, MD, USA), and 4 mM L-glutamine (Sigma-Aldrich S.p.a., Milan, Italy) at 37 °C in an atmosphere of 5% CO_2_. The cell culture was kept in an incubator (Thermo Scientific Hera Cell 240i, Waltham, MA, USA) at 37°C, 95% relative humidity, and 5% CO_2_ in air atmosphere. The complete growth medium (DMEM) was changed every two days. After being 75% confluent, the cells were washed with phosphate-buffered saline solution (PBS) (PAN Biotech, Aidenbach, Germany) to remove any unattached cells. The attached cells were harvested using a 1 mL 0.25% trypsin and 0.53 mM EDTA solution (Sigma-Aldrich S.p.a., Milan, Italy), seeded into 96-well plates at a density of 5 × 10^3^ cells per well, and incubated for 24 h to allow attachment before treatment with CdCl_2_ and MLT, Vitamin E, and Trolox.

#### 4.1.2. Preparation of Heavy Metal Solutions and Antioxidant Agents

Cadmium was administered as the water-soluble salt CdCl_2_. A stock solution of CdCl_2_ was prepared by dissolving 0.2283 g of powder in 10 mL of bidistilled sterile water under stirring, followed by filtration. The final concentration was 1 × 10^−1^ M. CdCl_2_ solutions at concentrations ranging from 1 to 100 μM were obtained by diluting the stock solution. A stock solution of MLT and Vitamin E was prepared by dissolving 0.240 g of powder in 1 mL of DMSO under stirring, while a stock solution of Trolox was prepared by dissolving 0.004 g in 1 mL of bidistilled sterile water. The final concentration was 1 × 10^−1^ M. Serial dilutions of the antioxidant agents were prepared to achieve the required final concentrations in each specific test. All the solutions were stored at 4 °C until use.

#### 4.1.3. Cytoprotective and Proliferative Effects

To assess the protective effect of antioxidant agents MLT, Vitamin E, and Trolox on Cd toxicity, SH-SY5Y, HCT116, and HepG2 cells were exposed to CdCl_2_, antioxidant agents, and combinations of MLT or Vitamin E or Trolox with CdCl_2_. The different cultures were divided into four experimental groups as follows: Group (1): control cells treated with 200 μL of each culture medium; Group (2): cells treated with (10–30 μM) of CdCl_2_ dissolved in medium culture (toxic treatment); Group (3): cells treated with 200 μL of culture medium containing MLT, or Vitamin E, or Trolox (100–300 μM); and Group (4): cells treated with MLT, or Vitamin E, or Trolox (100–300 μM) combined to CdCl_2_ (10–30 μM). The control groups consisted of SH-SY5Y, HCT116, and HepG_2_ cells processed in the same manner and incubated simultaneously as the treated groups [66] for 24 h of treatment.

#### 4.1.4. Cell Viability Evaluation by the Colorimetric MTT Assay

Cell line viability was determined via the 3-[4,5-dimethylthiazol-2-yl]-2,5-diphenyl tetrazolium bromide (MTT) reagent (Sigma-Aldrich S.p.a., Milan, Italy), which relies on the ability of mitochondrial oxidoreductases to convert soluble MTT into insoluble formazan in live cells [69,70]. In brief, SH-SY5Y, HCT116, and HepG2 cells were seeded at the density of 5 × 10^−4^ in a 96-well plate and then incubated with increasing concentrations of CdCl_2_, antioxidant agents, and combinations of MLT or Vitamin E or Trolox with CdCl_2_, respectively, (6 wells/concentration group plus 1 control group) for 24. Following this, the medium was taken out of the well, a solution of the MTT reagent—3-(4,5-dimetylthiazol-2-yl)-2,5-diphenyl tetrazolium bromide (5 mg/mL in PBS 1X)—was prepared, and 20 μL was added in 180 μL of the medium in the dark. After the incubation of cells with the MTT reagent for 3 h at 37 °C, the medium was removed from the wells. In each well, the dried formazan residue was reconstituted in 150 μL dimethyl sulfoxide (DMSO), and the mixture was kept warm for five minutes while being stirred. After that, the plates were placed into a multilabel microplate reader Victor 3 (PerkinElmer, Waltham, MA, USA), and the absorbance was measured using light with wavelengths λ 540 nm. The amount of formazan generated corresponded to the number of live cells. All MTT assays were performed in triplicate. Cell viability is expressed as a percentage of the control group (% control) calculated from the equation % control = Absorbance treatment/Absorbance control × 100%. Data are the mean percentages of viable cells vs. the respective controls.

#### 4.1.5. Measurement of Intracellular ROS Production

According to a slightly modified procedure already reported in the literature, the generation of ROS was determined using an oxidation-sensitive fluorescent probe, 2′,7′- dichlorodihydrofluorescein diacetate [23,28]. Briefly, HepG2 cells were seeded into a 96-black well plate for 24 h. The cells were treated simultaneously with the indicated concentrations of MLT, VitE, or Tr (100 and 300 µM) and with Cd^2+^ (10 or 30 µM) for 6 h or H_2_O_2_ (50 µM) for 30 min. Then, they were tested using a fluorescent probe (DCFH-DA, 25 µM), added in the dark, and incubated at 37 °C for 30 min. The formation of fluorescent dichlorofluorescein (DCF) due to oxidation of DCFH in the presence of ROS was read at 530 nm using a microplate reader Tecan Infinite M1000 Pro (Tecan, Cernuscosul Naviglio, Milan, Italy), and a DMSO medium was used for control cells.

### 4.2. Statistical Analysis

All experiments were performed in triplicate to ensure reproducibility. The results are presented as mean ± standard deviation (SD). The data were processed using GraphPad Prism version 9 (GraphPad Software Inc.; San Diego, CA, USA). Statistical significance was determined between untreated groups and treated groups (*p* < 0.0001) using a one-way analysis of variance (ANOVA) followed by Dunnett’s test. A value of *p* ≤ 0.05 was significant [71].

## 5. Conclusions

Cd toxicity is predominantly due to the production of oxidative stress across various tissue compartments. This study demonstrated that molecules with potent antioxidant activity, such as MLT, Vitamin E, and Trolox, are optimal candidates to counteract Cd-induced toxicity. This study highlights the potential cytoprotective effects of MLT, Vitamin E, and Trolox against Cd-induced toxicity in neuronal the SH-SY5Y, colorectal HCT 116, and hepatic HepG2 cell lines. Our results indicate that Vitamin E is the most effective antioxidant in mitigating the harmful effects of Cd, followed by MLT, which also showed significant protection, particularly at the highest Cd concentration of 30 µM. Furthermore, we first demonstrated that MLT, VitE, and Trolox were able to protect our cellular model from ROS production induced by stimulus with H_2_O_2_ in a dose-dependent manner. The same effect was demonstrated against Cd-induced ROS production. This research compares the antioxidant capabilities of MLT, Vitamin E, and Trolox under uniform experimental conditions. It highlights their respective efficacies and potential therapeutic applications. The findings underscore the significance of choosing the right antioxidant to combat heavy metal toxicity, with Vitamin E and its derivatives showing particular promise for therapeutic strategies against Cd exposure. However, further research is needed to elucidate the precise mechanisms underlying their protective effects and to evaluate their efficacy in vivo. Additionally, investigating the potential synergistic effects of these antioxidants with conventional treatments could provide valuable insights into developing comprehensive strategies for managing Cd toxicity. By assessing the efficacy of these antioxidants in preserving cellular viability and mitigating oxidative stress in the face of Cd exposure, this study aimed to contribute to the development of effective interventions to safeguard human health against the detrimental effects of environmental pollutants.

## Data Availability

The data are contained within this article and the Supplementary Materials.

## Supplementary Materials

The following supporting information can be downloaded at: https://www.mdpi.com/article/10.3390/ijms25158055/s1.
