# Supplementary material for: Evaluation of the Potential Cytoprotective Effect of Melatonin in Comparison with Vitamin E and Trolox against Cd2+-Induced Toxicity in SH-SY5Y, HCT 116, and HepG2 Cell Lines
Source: Int J Mol Sci. 2024 Jul 24;25(15):8055. doi: 10.3390/ijms25158055 (PMC11312335; doi:10.3390/ijms25158055)
Supplement: Supplementary file 1 [file ijms-25-08055-s001.zip › ijms-3067228-supplementary.pdf]

Table S1. Cell viability of SH-SY5Y, HCT 116 or HepG2 cells exposed to different concentrations of Cd^{2+} (1–100 µM) expressed as % cell viability ± SD.

Treatment groups	SH-SY5Y	HCT 116	HepG2
Cd^{2+} 1 µM	89.3 ± 1.7	71.8 ± 1.8	89.4 ± 1.5
Cd^{2+} 3 µM	55.9 ± 1.1	59.7 ± 1.3	80.2 ± 1.6
Cd^{2+} 10 µM	24.2 ± 1.5	47.6 ± 1.4	51.7 ± 2.1
Cd^{2+} 30 µM	16.8 ± 1.0	30.6 ± 1.1	8.3 ± 0.4
Cd^{2+} 100 µM	15.2 ± 1.1	16.2 ± 2.0	4.2 ± 0.7

Table S2. Cell viability of SH-SY5Y, HCT 116 or HepG2 cells exposed to 100 or 300 µM of MLT, VitE or Trolox, expressed as % cell viability ± SD.

Treatment groups	SH-SY5Y	HCT 116	HepG2
MLT 100 µM	78.5 ± 2.2	68.9 ± 2.1	78.8 ± 0.9
MLT 300 µM	74.9 ± 0.7	71.6 ± 1.4	63.6 ± 1.2
VitE 100 µM	71.3 ± 1.2	59.7 ± 1.5	101.8 ± 0.9
VitE 300 µM	79.6 ± 0.9	66.1 ± 2.2	109.9 ± 2.2
Trolox 100 µM	76.7 ± 1.7	69.9 ± 1.9	77.0 ±1.5
Trolox 300 µM	80.3 ± 2.1	73.5 ± 2.0	62.2 ± 0.8

Table S3. Cell viability of SH-SY5Y, HCT 116 or HepG2 cells exposed to 10 µM of Cd^{2+} alone or co-treated with Cd^{2+} 10 µM and MLT, VitE or Trolox (100 or 300 µM) expressed as % cell viability ± SD.

Treatment groups	SH-SY5Y	HCT 116	HepG2
Cd^{2+} 10 µM	24.3 ± 2.3	46.7 ± 0.8	51.7 ± 2.1
Cd^{2+} 10 µM + MLT 100 µM	41.8 ± 1.7	46.7 ± 1.3	51.7 ± 1.8
Cd^{2+} 10 µM + MLT 300 µM	47.3 ± 1.9	30.4 ± 0.8	47.4 ± 1.4
Cd^{2+} 10 µM + VitE 100 µM	58.2 ± 1.4	42.0 ± 1.2	30.4 ± 2.0
Cd^{2+} 10 µM + VitE 300 µM	52.4 ± 1.9	51.1 ± 0.4	80.7 ± 0.4
Cd^{2+} 10 µM + Trolox 100 µM	46.9 ± 2.0	47.2 ± 2.0	67.8 ± 1.9
Cd^{2+} 10 µM + Trolox 300 µM	58.9 ± 2.2	45.6 ± 0.9	43.0 ± 2.2

Table S4. Cell viability of SH-SY5Y, HCT 116 or HepG2 cells, exposed to 30 μM of Cd^{2+} alone or co-treated with Cd^{2+} 30 μM and MLT, VitE or Trolox (100 or 300 μM) expressed as % cell viability ± SD.

Treatment groups	SH-SY5Y	HCT 116	HepG2
Cd^{2+} 30 μM	16.8 ± 1.3	31.6 ± 0.8	8.3 ± 1.2
Cd^{2+} 30 μM + MLT 100 μM	21.7 ± 2.2	41.6 ± 1.0	66.2 ± 2.1
Cd^{2+} 30 μM + MLT 300 μM	60.9 ± 2.4	31.6 ± 2.2	7.6 ± 1.1
Cd^{2+} 30 μM + VitE 100 μM	63.3 ± 1.9	58.6 ± 0.9	27.3 ± 2.0
Cd^{2+} 30 μM + VitE 300 μM	80.7 ± 1.6	60.2 ± 1.5	35.2 ± 1.6
Cd^{2+} 30 μM + Trolox 100 μM	77.7 ± 2.2	71.4 ± 1.4	78.6 ± 1.2
Cd^{2+} 30 μM + Trolox 300 μM	97.7 ± 1.6	67.4 ± 1.8	47.4 ± 1.7

Table S5. DCFH oxidation in HepG2 cells exposed to 50 μM of H_2O_2 alone or co-treated with H2O2 50 μM and MLT, VitE or Trolox (100 or 300 μM) expressed as % of fluorescence intensity ± SD.

Treatment groups	HepG2
H_2O_2 50 μM	225.3 ± 1.0
H_2O_2 50 μM + MLT 100 μM	220.9 ± 2.0
H_2O_2 50 μM + MLT 300 μM	164.3 ± 1.6
H_2O_2 50 μM + VitE 100 μM	194.4 ± 2.2
H_2O_2 50 μM + VitE 300 μM	169.0 ± 0.9
H_2O_2 50 μM + Trolox 100 μM	219.4 ± 2.1
H_2O_2 50 μM + Trolox 300 μM	149.0 ± 1.7

Table S6. DCFH oxidation in HepG2 cells exposed to 10 μM of Cd^{2+} alone or co-treated with Cd^{2+} 10 μM and MLT, VitE or Trolox (100 or 300 μM) expressed as % of fluorescence intensity ± SD.

Treatment groups	HepG2
Cd^{2+} 10 μM	301.5 ± 2.2
Cd^{2+} 10 μM + MLT 100 μM	295.7 ± 2.4
Cd^{2+} 10 μM + MLT 300 μM	280.8 ± 1.8
Cd^{2+} 10 μM + VitE 100 μM	264.0 ±1.6
Cd^{2+} 10 μM + VitE 300 μM	254.3 ± 2.2
Cd^{2+} 10 μM + Trolox 100 μM	236.7 ± 2.3

Cd^{2+} 10 μM + Trolox 300 μM	159.9 ± 1.9

Table S7. DCFH oxidation in HepG2 cells, exposed to 30 μM of Cd^{2+} alone or co-treated with Cd^{2+} 30 μM and MLT, VitE or Trolox (100 or 300 μM) expressed as % of fluorescence intensity ± SD.

Treatment groups	HepG2
Cd^{2+} 30 μM	389.1 ± 2.1
Cd^{2+} 30 μM + MLT 100 μM	386.6 ± 1.9
Cd^{2+} 30 μM + MLT 300 μM	301.4 ± 1.7
Cd^{2+} 30 μM + VitE 100 μM	351.1 ± 2.2
Cd^{2+} 30 μM + VitE 300 μM	279.0 ± 1.4
Cd^{2+} 30 μM + Trolox 100 μM	226.2 ± 1.9
Cd^{2+} 30 μM + Trolox 300 μM	196.5 ± 2.2
